# Caspofungin for Primary Antifungal Prophylaxis in Acute Myeloid Leukemia: A Real-Life Study from an Academic Center

**DOI:** 10.3390/cancers17132184

**Published:** 2025-06-28

**Authors:** Francesco Grimaldi, Mara Memoli, Simona Avilia, Carlangela Causa, Maria Luisa Giannattasio, Italia Conversano, Dario Lisi, Daniela D’Angelo, Raffaella Iannotta, Nicola Schiano Moriello, Giulio Viceconte, Emanuela Zappulo, Ivan Gentile, Marco Picardi, Fabrizio Pane

**Affiliations:** 1 Department of Clinical Medicine and Surgery, Hematology Division, University of Napoli “Federico II”, 80131 Naples, Italy; mara.memoli@unina.it (M.M.); simona.avilia@unina.it (S.A.); carlangela_causa@virgilio.it (C.C.); marial.giannattasio@studenti.unina.it (M.L.G.); i.conversano@studenti.unina.it (I.C.); dario.lisi@unina.it (D.L.); daniela.dangelo@unina.it (D.D.); ra.iannotta@studenti.unina.it (R.I.); marco.picardi@unina.it (M.P.); fabrizio.pane@unina.it (F.P.); 2 Department of Clinical Medicine and Surgery, Infectious Diseases Division, University of Napoli “Federico II”, 80131 Naples, Italy; nicola.schianomoriello@unina.it (N.S.M.); giulio.viceconte@unina.it (G.V.); emanuela.zappulo@unina.it (E.Z.); ivan.gentile@unina.it (I.G.)

**Keywords:** caspofungin, antifungal prophylaxis, IFI, acute myeloid leukemia, venetoclax, midostaurin

## Abstract

**Background:** Invasive fungal infections (IFIs) are a major complication in patients with acute myeloid leukemia (AML), particularly during chemotherapy-induced neutropenia. Posaconazole is the standard drug for primary antifungal prophylaxis (PAP), but its use is limited by oral bioavailability and CYP3A4 interactions. **Study Objective:** This study aims to evaluate the clinical efficacy and safety of intravenous caspofungin versus oral posaconazole as PAP in AML patients during their first cycle of chemotherapy and assess their subsequent impact on clinical outcomes. **Methods:** A retrospective, monocentric study was conducted on 75 consecutive AML patients treated at the Federico II University Medical School of Naples, Italy (2021–2025). Patients received either caspofungin or posaconazole as PAP based on the drug–drug interaction risk or clinical conditions. IFIs were diagnosed using EORTC/MSG criteria. Logistic and Cox regression models were used to assess risk factors and overall survival (OS). **Results:** IFI incidence was 13.3% overall (9.4% proven/probable). No significant difference was found between the caspofungin and posaconazole groups (six vs. four IFIs; *p* = 0.878). Post-chemotherapy refractory AML (OR = 11.9; *p* = 0.003) and liver disease (OR = 30.4; *p* = 0.004) independently predicted IFI development. Median OS did not significantly differ in patients receiving caspofungin versus posaconazole (29.3 vs. 32.1 months, *p* = 0.6). **Conclusions:** Caspofungin appears clinically comparable to posaconazole for PAP in AML during the induction phase, especially when azole use is contraindicated. Prospective studies are warranted to refine prophylactic strategies in the era of new AML therapies.

## Simple Summary

Patients with acute myeloid leukemia often deal with serious infections due to a drop in neutrophil cell count caused by chemotherapy. One of the most dangerous types of infection in this setting is invasive fungal infections, which can be difficult to prevent and treat. This study examined two different strategies to prevent fungal infections during the first cycle of chemotherapy in acute myeloid leukemia: posaconazole, an oral antifungal drug, and caspofungin, an antifungal agent given through intravenous injection. In this comparison, both treatments were similarly effective in preventing fungal infections and were generally well tolerated. However, patients with liver problems or with refractory leukemia were found to be at a higher risk of developing fungal infections. Finally, caspofungin proved to be just as effective as posaconazole, particularly for patients unable to take oral medications or those at risk of drug-to-drug interactions when undergoing chemotherapy. These findings may help clinicians choose safer and more effective prevention strategies for patients undergoing treatment for acute myeloid leukemia. Further studies, however, will be needed to better understand which patients could benefit the most from each of these approaches.

## 1. Introduction

Invasive fungal infections (IFIs) are a well-known complication of patients affected by acute myeloid leukemia (AML) [1], and chemotherapy-related profound neutropenia is nowadays still considered the major risk factor for their development [2]. The expected incidence of probable/proven IFIs changes during the treatment phase [3] and is estimated to be about 10% during remission induction chemotherapy [4], which decreases to a rate of 1.5–2.5% during consolidation chemotherapy [5]. Therefore, induction chemotherapy for AML remains the critical period in which the burden of IFIs is still of great concern, with a potential negative impact on outcome [6] and a mortality risk still accounting for up to 30% of cases [7].

A reduction in IFI incidence and mortality has been reported after the introduction of systematic antifungal prophylaxis, as shown in several studies [8,9] and meta-analyses [10,11], particularly with the advent of posaconazole [12]. Posaconazole is an oral triazolic agent, nowadays formally recommended for primary antifungal prophylaxis (PAP) during induction chemotherapy for AML, that shows particularly strong activity as a CYP3A4 inhibitor [13]. As an oral drug, its administration can be limited by patient conditions such as intercurrent chemotherapy-related gastrointestinal mucositis [14] or poor performance status due to disease or treatment complications. Moreover, the introduction of new, targeted drugs able to interact with CYP3A4 in AML practice, such as venetoclax [15] and midostaurin [16], recently posed some issues on the use of posaconazole in AML patients. For venetoclax, a dose reduction of 50% to 75% during coadministration with posaconazole has been suggested by an EHA (European Hematology Association) expert panel [17], mainly based on pharmacokinetics studies [18]. For midostaurin, conflicting recommendations on which antifungal agent to coadminister during induction chemotherapy are available, leading to a real clinical dilemma [19] on which PAP to choose in this situation. Therefore, alternative antifungal prophylactic strategies in patients treated with midostaurin or VEN-based regimens, or in patients unable to consume oral drugs due to impaired clinical conditions, remain desirable and warranted.

Caspofungin is a member of the echinocandin family that inhibits the enzyme 1,3-β-D-glucan synthase, disrupting fungal cell-wall synthesis. It has a broad spectrum of activity against Candida and Aspergillus species [20] and is known for its favorable safety profile, largely given by minimal drug–drug interactions [21]. Importantly, caspofungin is administered intravenously, which ensures consistent plasma levels regardless of gastrointestinal function or patient conditions, with efficacy not compromised by mucositis, food effects, or concurrent medications that affect drug metabolism. In prospective clinical trials, caspofungin has been shown to be effective and safe for the treatment of IFIs in critically ill patients [22] and in patients with hematological cancer [23,24]. Moreover, good results have been observed in AML both as empirical therapy during febrile neutropenia [25] and as primary prophylaxis in patients undergoing hematopoietic stem cell transplantation (HSCT) [26]. Finally, two randomized trials and one meta-analysis showed good efficacy of caspofungin when used preemptively [27] in the antifungal treatment of high-risk neutropenic patients with AML, when used as the prophylaxis [28] of IFIs among children and young adults with acute leukemia, and when compared with other azoles in neutropenic AML patients [29]. Reflecting this growing evidence, clinical guidelines [30,31] have started to endorse echinocandins for antifungal prophylaxis, and in the latest ECIL-10 (European Conference on Infections in Leukaemia) recommendations [32] for AML, echinocandins were upgraded from a previous C-II level to a B-II level, providing evidence for the use of PAP. Consequently, caspofungin and other echinocandins are now considered suitable alternative agents for primary prophylaxis against IFIs in AML [33].

To further clarify the role of caspofungin in AML, we conducted a real-life retrospective study evaluating the incidence of IFIs in patients who received caspofungin in place of posaconazole as PAP.

## 2. Materials and Methods

### 2.1. Study Design

A real-life retrospective monocentric study was conducted at the Hematology Division of the Federico II University Medical School of Naples, Italy, in patients who received antifungal prophylaxis in the context of the first cycle of chemotherapy for AML. The primary objective of the study was to document the incidence of possible/probable/proven IFIs occurring in AML patients receiving caspofungin instead of posaconazole as PAP to avoid drug–drug interactions (if treated with intensive chemotherapy and midostaurin or with a venetoclax-based regimen) or due to patient conditions that impaired oral drug consumption. The secondary objectives were to assess the safety of the different prophylaxis strategies and to identify any potential risk factors associated with IFI diagnosis. The tertiary objective of the study was to evaluate the impact of IFI diagnosis, the type of antifungal prophylaxis, patient demographics, and disease-related factors on overall survival (OS).

### 2.2. Definitions

IFIs were diagnosed according to updated EORTC/MSG criteria [34] and categorized as possible, probable, or proven. The classification of AML diagnosis was made according to the 2022 World Health Organization (WHO2022) [35] and 2022 International Consensus Classification for myeloid neoplasm (ICC2022) [36] criteria, while classification of AML risk and response criteria were made according to European Leukemia Net (ELN) 2022 risk stratification [37]. The duration of neutropenia was defined as the number of days with an absolute neutrophil count (ANC) below 500/mmc.

### 2.3. Diagnostic Work-Up

In all patients who planned to receive AML treatment, galactomann antigen (GM) and Beta-D-glucan (BD) detection tests were performed once weekly and twice weekly in patients with fever. GM antigen levels were expressed as an optical index (calculated by dividing the blood sample optical density by the kit’s test control). An index ≥ 0.5 in serum was considered positive. BD levels were assessed by turbidimetric (Fungitell) assays and measured in picograms per milliliter (pg/mL). In neutropenic patients, antibacterial prophylaxis consisting of levofloxacin 500 mg once daily was administered to all patients. Topical antifungal therapy with polyene agents and antiviral prophylaxis with acyclovir were also used depending on patient characteristics. At fever onset, patients were tested with blood cultures and chest X-rays. Empirical broad-spectrum antibiotic treatment began and was escalated in case of the persistence or recurrence of a fever. Moreover, a CT scan was performed in case of a persistent fever persisting for ≥72 h or in case of a recurring fever, particularly in patients with suggestive infective symptoms (i.e., dyspnea or diarrhea). Switching from caspofungin to a systemic antifungal therapy with other antifungal agents was considered after multidisciplinary discussion with the infectious disease team.

### 2.4. Statistical Analysis

Descriptive statistics were calculated for all variables of interest. Categorical variables were summarized through frequencies and percentage values, while continuous variables were summarized through median values and their relative ranges. Comparisons between groups were tested by the Chi-square test and the Wilcoxon–Mann–Whitney U-test. Correlation analyses were performed by Spearman’s rank correlation ρ testing, while univariate and multivariate logistic regression models were used to estimate the impact of covariates on categorical events. Overall survival (OS) analysis was carried out by the Kaplan–Meier product-limit method and by Cox proportional hazard regression models. OS was defined as the time from the first diagnosis to the death of the patient or, if censored, the last contact with the patient. The log-rank test was used to show any statistically significant difference between subgroups. For estimating the risk of specific events, odds ratios (ORs) and hazard risks (HRs), with relative 95% confidence intervals (95% CIs), were estimated for each covariate using the logistic regression model or Cox analysis. In multivariate models, the enter method analysis, including all clinically relevant variables, was used to evaluate interaction between covariates. A *p*-value < 0.05 was considered statistically significant in the results. Statistical analyses were carried out using R package software (R Statistical Software v4.5.0; R Core Team 2021).

## 3. Results

### 3.1. Study Population

Data from 75 patients (40 females (53%); 35 males (47%)) treated for AML at the Hematology Division of Federico II University Hospital from January 2021 through January 2025 were retrospectively collected and analyzed. The median follow-up time of the patients was 21 months (range 3–42). The median age was 61 years (range: 26–75), with no significant differences in the male/female ratio. The median number of comorbidities per patient was 1 (range: 0–4), with type II diabetes (16 patients, 21%) and cardiovascular disease (12 patients, 16%) as the more frequent comorbidities. According to WHO2022 and ICC2022 classifications, the diagnosis of AML with defining genetic abnormalities was found in 40 (53.3%) cases, AML with myelodysplastic syndrome gene mutations (AML MDS-related) in 12 (16%) cases, AML defined by differentiation in 8 (11%) cases, AML secondary to previous myeloproliferative neoplasms (AML post-MPN) in 7 (9.3%) cases, therapy-related AML in 3 (4%) cases, and finally, MDS/AML with a *TP53* mutation in 5 (6.7%) cases. According to ELN2022 classification, 28 (37%) patients had a low-risk disease, 25 (33%) patients an intermediate-risk disease, and 22 (29%) patients a high-risk disease. Looking at *NPM1* and *FLT3* statuses, 26 (34%) patients had *NPM1* mutations, and 19 (25%) patients had *FLT3* mutations. AML chemotherapy consisted of IDA-Flag (fludarabine, cytarabine, idarubicin, and filgrastim) in 4 (5%) patients, CPX-351 in 11 (14%) patients, 3 + 7 (daunorubicin 3 days + cytarabine 7 days) in 16 (21%) patients, 3 + 7 + gemtuzumab ozogamicin (3 + 7 + GO) in 15 (20%) patients, 3 + 7 + midostaurin in 12 (16%) patients, and hypomethilating agents (HMAs) + venetoclax (HMAs/Ven) in 17 (24%) patients (4 decitabine and 13 azacitidine). Post-chemotherapy neutropenia was observed in all patients, with a median duration of 22 days (range: 12–131).

### 3.2. PAP Strategy

Antifungal prophylaxis was delivered during the first cycle of chemotherapy in all patients. Standard prophylaxis with posaconazole was used in 42 patients (56%), while caspofungin was used as antifungal prophylaxis in 33 (44%) patients. Caspofungin was the first antifungal of choice to avoid drug-to-drug interactions with venetoclax or midostaurin in 20 patients (61%), while in the other 13 patients (39%), caspofungin was preferred to posaconazole as PAP mainly due to patient conditions that impaired oral posaconazole administration (i.e., deteriorated performance status, oral mucositis, and gastrointestinal symptoms). According to drug schedules, posaconazole was administered orally at 300 mg once daily, with the first two doses given every 12 h, while caspofungin was given intravenously (IV) 50 mg once daily, with the first dose at 70 mg. The median duration of PAP was 22 days (range: 13–68) for posaconazole and 26 days (range: 12–131) for caspofungin.

### 3.3. Characteristics of Posa-Group Versus Caspo-Group

No significant differences in terms of demographics (age, sex, burden of comorbidities, and most prevalent comorbidity), AML type, ELN2022 risk, the presence of *NPM1* or *FLT3* mutations, and the duration of neutropenia were observed across the two groups (caspofungin vs. posaconazole prophylaxis). As expected by the study design, a higher proportion of *FLT3* patients were treated with midostaurin (*p* < 0.005) in the caspofungin group, while a significantly higher proportion of patients in the posaconazole group received alternative induction regimens such as 3 + 7 ± GO (*p* < 0.001). Patient characteristics with significant differences between the two prophylaxis groups are summarized in Table 1.

### 3.4. IFI Incidence, Association with Type of Prophylaxis, Clinical Course, and Safety

**Figure 1:**
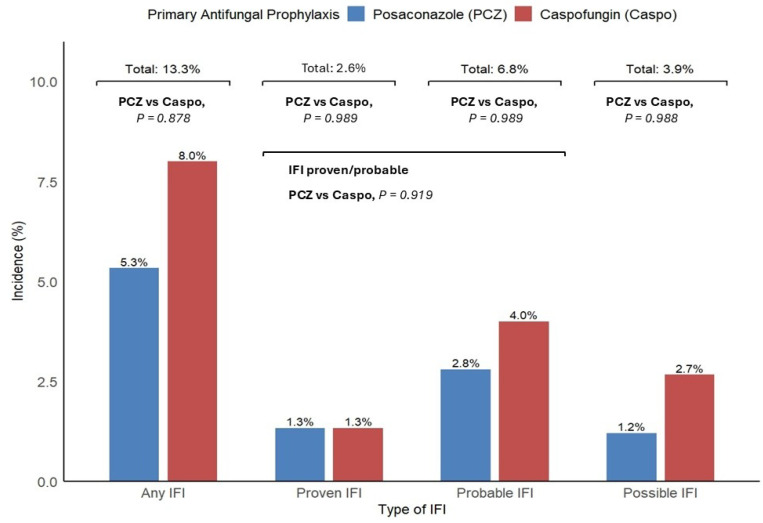
IFI and subtype incidence as a percentage of the total population (n = 75).

A total of 10 episodes of IFIs were diagnosed in the study, for an overall incidence of 13,3%. IFI diagnosis occurred after intensive 3 + 7±midostaurin chemotherapy in six cases and after venetoclax-based therapy in the remaining four. According to EORTC/MSG criteria, IFIs were classifiable as proven, probable, and possible in two (2.6%), five (6.8%), and three (3.9%) cases, respectively, for an incidence of 9,4% for proven and probable infections (seven patients). The median time from chemotherapy start to the diagnosis of IFI was 25 (range: 18–53) days. When IFIs were grouped according to antifungal prophylaxis, an increased but not significant number of IFIs was observed in the caspofungin prophylaxis group (six vs. four; *p* = 0.878). Even when possible IFIs were excluded, and only proven and probable IFIs were considered in the analysis, the difference between the two prophylaxis groups remained not significant (four vs. three; *p* = 0.919), but still with a majority of cases in the caspofungin group (Figure 1).

*Geotrichum* spp. and *Saprochaete capitata* were responsible for the two cases of proven IFIs. *Geotrichum* was isolated from blood cultures of a patient in the caspofungin prophylaxis group. After switching to systemic antifungal therapy, a head CT scan performed for neurological symptoms showed radiological findings compatible with fungal CNS disease. *Saprochaete capitata* was isolated from blood cultures and a liver biopsy of a patient in the posaconazole prophylaxis group. In both patients, these fungal infections were resistant to systemic second-line therapy with liposomal B amphotericin (L-AmB) and ultimately led to patient death, resulting in a global fungal death rate of 2% (2/75).

In five cases (three in the caspofungin group and two in the posaconazole group), the diagnosis of IFIs was compatible with probable lung aspergillosis, with typical radiological findings by lung CT scans and serum GM elevation (median GM value: 1.1; range: 0.93–2.28). Overall, the lungs remained as the main site of infection in the caspofungin group, with five (71%) cases recorded (three probable + two possible IFIs). The diagnosis of possible IFIs was made in two neutropenic patients in the caspofungin group due to persistent fever with clinical respiratory symptoms, the absence of specific microbiological isolates, and a positive CT scan showing lung macronodules, which were ultimately compatible with possible lung aspergillosis. In one neutropenic patient in the posaconazole group, a diagnosis of possible candidiasis was made due to liver and spleen micronodules with target signs detected by ultrasound, which showed them to be radiologically compatible with mycotic localization, in the absence of significant BD or GM serum-level increases, and without any microbiological isolates.

In all cases of probable/possible IFIs, an antifungal switch therapy was performed. Isavuconazole was the drug of choice in four cases of probable aspergillosis. Only one patient with probable lung aspergillosis was treated with L-AmB, and L-AmB was used in the other three cases with possible IFIs. A radiologically documented response to newer antifungal therapy was observed in 7 out of 10 (70%) cases, allowing continuation of AML treatment. Only one patient diagnosed with possible lung IFI did not improve with L-AmB treatment, suggesting the presence of an alternative, non-fungal infection that was not identified.

Overall, PAP was safe and well tolerated with both drugs. No particular toxicities were observed with caspofungin, while grade 1–2 liver enzyme elevations, which did not determine drug suspension, were observed in 15 (33%) patients receiving posaconazole.

### 3.5. Risk Factors Associated with IFI Incidence and Survival Analysis

**Figure 2:**
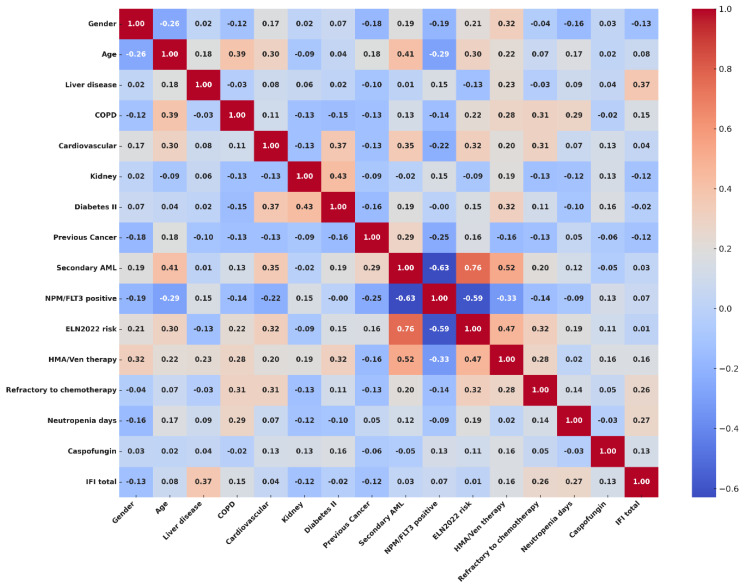
Correlation matrix showing interactions between covariates and the risk of IFIs.

To explore the risk factors potentially related to the incidence of IFI in our population, we performed an exploratory analysis with Spearman’s rank correlation test. Categorical variables included in the analysis were gender (male vs. female), the presence or lack thereof of clinically significant comorbidities—such as liver disease (i.e., severe liver steatosis), chronic obstructive pulmonary disease (copd), cardiovascular comorbidities, kidney function impairment (i.e., moderate-to-severe renal failure), and type II diabetes—a history of previous cancer (yes vs. no), a diagnosis according to WHO and ICC2022 (comparing secondary AML vs. de novo AML), positivity for NPM or FLT3 mutations (positive vs. negative), ELN2022 risk (high vs. low/intermediate), type of chemotherapy (HMA/VEN vs. intensive regimens), and disease status after treatment (refractory vs. responding disease). The age of the patients and the number of days of severe neutropenia were included in the model as continuous variables. Strong positive correlations were found with liver disease (ρ = 0.373; CI95%, 0.033–0.676), days of severe neutropenia (ρ = 0.257; CI95%, 0.087–0.428), and disease status (ρ = 0.269; CI95%, 0.064–0.539) after chemotherapy. Mild correlations with IFIs were observed for the presence of COPD (ρ = 0.150; CI95%, 0.122–0.414), exposure to an HMA/Ven regimen (ρ = 0.162; CI95%, 0.087–0.421), and usage of caspofungin as an antifungal prophylaxis (ρ = 0.126; CI95–0.131–0.360). The complete results of the correlation analyses are available in the Supplemental Results (S1), and a correlation matrix showing the interactions between covariates and correlations with IFIs is visualized in Figure 2.

**Figure 3:**
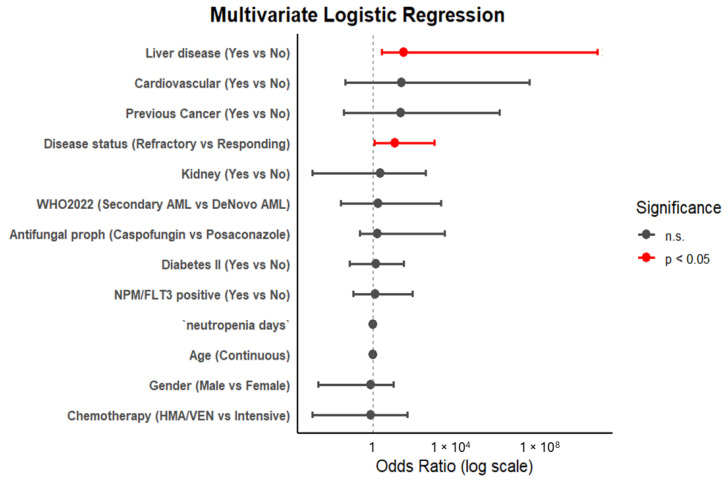
Forest plot with risk factors for proven or probable IFI.

To further assess the possible independent association of clinical factors with a diagnosis of IFI and to assess the interactions between covariates, we performed a univariate and multivariate logistic regression analysis. An initial exploratory analysis that took into account all the variables, with incidences of proven, probable, and possible IFIs, is reported in the Supplemental Results (S2). Considering sample dimensions and the number of events, we then focused our analysis only on the group of proven and probable IFIs. In the univariate model, only liver disease (*p* = 0.01; CI95%, 1.53–56.79), the duration of neutropenia, and refractory disease after chemotherapy (*p* = 0.05; CI95%, 0.86–26.17) were shown to be statistically significant (see Supplemental Results (S3) for the complete results). However, in multivariate logistic analysis, only liver disease (OR = 30.4; *p* = 0.004) and refractory disease status after chemotherapy (OR = 11.9; *p* = 0.003) were confirmed as significant factors in IFI incidence. The impact of COPD, exposure to an HMA/Ven regimen, and usage of caspofungin were not confirmed in univariate or multivariate analyses (see Supplemental Results (S4) for the complete results). A forest plot analysis, visualizing multivariate logistic regression results, is available in Figure 3.

**Figure 4:**
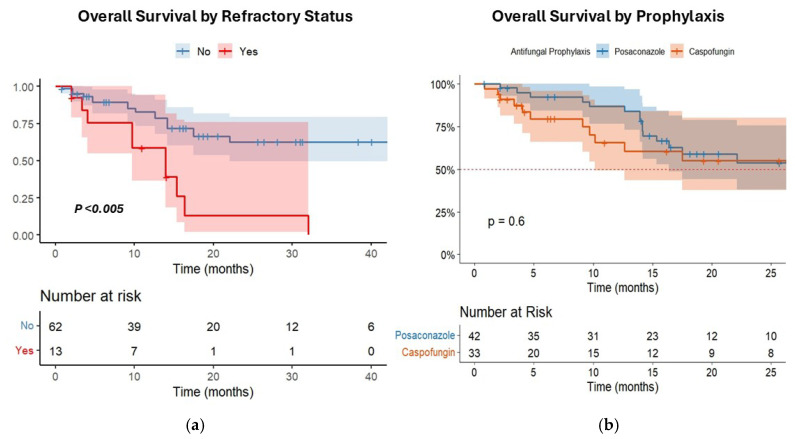
Kaplan–Meier diagram showing patients’ survival by (**a**) disease status after induction chemotherapy (refractory disease vs. responding disease) and (**b**) type of antifungal prophylaxis (posaconazole vs. caspofungin).

In survival analysis, the OS of patients with refractory disease after treatment was significantly inferior to patients with responding disease (median OS = 13.9 months [CI95% 9.5–13.7] vs. 34.4 months [CI95% 17.5–42.1], *p* < 0.005) (Figure 4a). Conversely, no significant difference in OS was observed according to the type of prophylaxis (caspofungin vs. posaconazole) (median OS = 29.3 months [CI95% 19.5–35.7] vs. 32.1 months [CI95% 17.5–33.1], *p* = 0.6) (Figure 4b).

Univariate Cox regression showed that classical risk factors such as age, type of AML, FLT3 status, type of chemotherapy, duration of neutropenia, disease status after treatment, HSCT, and diagnosis of IFIs have a significant impact on survival. However, only age and disease status retained their significance in the multivariate analysis model (see Table 2). Considering that refractory disease significantly influenced OS in our cohort, we finally analyzed the impact of different PAP strategies in patients with refractory disease status. Interestingly, a significant difference emerged between patient groups in favor of posaconazole (posaconazole = 15.3 months [CI95% 13.9–NA] vs. caspofungin = 4.03 months [CI95% 3.3–10.1], *p* < 0.001) (see Supplemental Results (S5)).

## 4. Discussion

The evolving therapeutic landscape of AML, marked by the integration of targeted agents such as venetoclax and midostaurin, has prompted renewed attention toward antifungal prophylaxis strategies that minimize pharmacologic interactions. Moreover, several guidelines [30,31] and recently updated ECIL-10 recommendations [32] have enforced the role of echinocandins as primary antifungal agents in hematological patients. Therefore, alternative strategies for antifungal prophylaxis are now considered for AML.

Our study supports the use of caspofungin as a viable alternative to posaconazole, particularly in clinical contexts where oral posaconazole administration is impractical due to patient conditions or contraindicated due to drug–drug interactions. The overall incidence of IFIs (13.3%) observed in our study is consistent with data reported in the literature [1,2,3,4] and remained comparable between prophylaxis groups with no statistical difference, as well as with no significant correlation between caspofungin and IFIs in logistic regression analysis, supporting the non-inferiority of echinocandins as PAP in AML. Although a numerically higher frequency of probable lung aspergillosis was observed in patients receiving caspofungin, probably due to the narrowed efficacy on mold infections compared to azoles, this condition did not translate into a worse outcome and reflects the ability of second-line antifungal therapy to provide good clinical responses in this setting. Moreover, in our real-life setting, the 70% response rate observed with Isavuconazole and L-AmB confirms the good efficacy of these drugs in managing lung [38] and liver [39] IFIs, as reported in the literature.

Beyond the choice of prophylactic agent, key risk factors for IFIs that can inform clinical practice are evidenced in our study. Correlation analysis and multivariate logistic regression identified pre-existing liver dysfunction and refractory leukemia (failure to achieve remission) as the strongest independent predictors of IFI development. Liver failure has already been associated with an increased risk for IFIs in non-hematological patients [40]. Even AML patients with significant hepatic comorbidities might present a markedly higher likelihood of breakthrough infection, due to their intrinsic vulnerability or altered drug metabolism, although this data needs to be confirmed in larger samples, given the retrospective nature of our study. Differently, the evidence that persistent disease and prolonged neutropenia were associated with a higher risk of IFIs in our population confirms previously well-reported data and reflects the crucial impact of disease control on infection susceptibility. Other commonly cited factors such as chemotherapy regimen intensity (e.g., cytarabine/anthracycline ± midostaurin or GO vs. hypomethylating agents plus venetoclax) and the presence of moderate comorbidities (like COPD, cardiovascular disease, or diabetes) did not independently predict IFIs in our analysis, even if some underestimation could be derived from the retrospective nature and the small size of the study.

Survival analysis confirmed that IFI occurrence did not independently influence OS once other prognostic variables were accounted for, supporting the notion that most IFIs, when promptly diagnosed and treated, do not compromise AML outcomes, given the therapeutic options available today. Only infections caused by uncommon molds (i.e., proven IFIs by *Saprochaete capitata* and *Geotrichum* spp.) led to specific fungal mortality. Both IFIs occurred as breakthrough infections during antifungal prophylaxis, as previously described [41]. *Geotrichum* spp. showed limited susceptibility to voriconazole, while *S. capitata* was intrinsically resistant to echinocandins. Apart from this, clear predisposing risk factors were not evident, highlighting the need for continued vigilance and aggressive management in such cases, where an effective prophylactic agent is still missing [42]. Interestingly, in patients with refractory disease, a significant OS difference emerged based on the prophylaxis strategy, favoring posaconazole treatment. Although this data needs to be confirmed in larger studies, the difference may reflect a more robust mold coverage of posaconazole in the setting of prolonged neutropenia, which is more likely to happen in non-responding patients. In line with this, the finding that the duration of neutropenia remained a significant factor only in univariate analysis enforces the idea that neutropenia may exert its prognostic impact not in a global population but only in specific settings (i.e., refractory disease).

Our study is inherently limited by its retrospective design, as well as the numerosity and heterogeneity of the study population, which includes patients receiving both intensive and less-intensive venetoclax-based treatment regimens for AML (where the actual, real risk for IFIs remains debated) [43,44]. However, these therapeutic approaches reflect a real-world context in which the optimal strategy for antifungal prophylaxis needs to be refined.

## 5. Conclusions

Despite limitations given by the retrospective nature of the study, and the relatively smaller sample size, our findings suggest that caspofungin is a clinically comparable alternative to posaconazole for primary antifungal prophylaxis in AML patients, particularly in scenarios where azoles use is contraindicated. However, patients with prolonged neutropenia and refractory disease remain the most vulnerable population where probably azoles remain the standard of care in terms of antifungal prolhylaxis.

Prospective studies on larger number of patients will be needed to better define individual risk profiles and guide personalized antifungal prophylactic strategies in this population.

## Figures and Tables

**Table 1:** Patient characteristics.

	Caspofungin	Posaconazole	*p*
**Male/female, n**	16/17	19/23	0.928
**Age, median (range)**	61 (26–75)	60 (31–74)	0.928
**Comorbidities, median (range)**	1 (0–4)	1 (0–4)	0.919
**Type II diabetes, n (%)**	9 (27%)	7 (16%)	0.619
**Cardiovascular disease, n (%)**	7 (21%)	5 (11%)	0.616
**AML with defining genetic abnormalities, n (%)**	20 (61%)	22 (54%)	0.787
**AML MDS-related, n (%)**	5 (15%)	7 (16%)	0.928
**AML defined by differentiation, n (%)**	2 (6%)	4 (9.5%)	0.919
**AML post-MPN, n (%)**	3 (9%)	4 (9.5%)	0.919
**MDS/AML *TP53* mutation, n (%)**	2 (6%)	3 (7%)	0.919
**Therapy-related AML, n (%)**	1 (3%)	2 (4%)	0.919
**ELN2022 low, n (%)**	7 (22%)	15 (35%)	0.204
**ELN2022 intermediate, n (%)**	13 (39%)	12 (30%)	0.765
**ELN 2022 high, n (%)**	13 (39%)	15 (35%)	0.760
***NPM1* pos, n (%)**	14 (42%)	12 (30%)	0.606
***FLT3* pos, n (%)**	11 (33%)	7 (16%)	0.325
**IDA-Flag, n (%)**	1 (3%)	3 (7%)	0.628
**CPX-351, n (%)**	4 (12%)	7 (16%)	0.675
**3 + 7, n (%)**	1 (3%)	15 (36%)	<0.001
**3 + 7+GO, n (%)**	5 (15%)	10 (25%)	0.406
**3 + 7+midostaurin, n (%)**	12 (36%)	0	<0.005
**HMA/venetoclax, n (%)**	10 (31%)	7 (16%)	0.411
**Days of neutropenia,** **median (range)**	22 (13–68)	26 (12–131)	0.928
**Refractory status** **after chemotherapy, n (%)**	6 (18%)	7 (16%)	0.919

AML = acute myeloid leukemia; MDS = myelodysplastic syndrome; MPN = myeloproliferative neoplasm; ELN = European Leukemia Net; GO = gemtuzumab ozogamicin; 3 + 7 = daunorubicin, cytarabine; IDA-Flag = fludarabine, cytarabine, idarubicin, and filgrastim; HMAs = hypomethylating agents.

**Table 2:** Cox univariate and multivariate analysis for factors impacting overall survival.

Univariate Analysis				
Covariate	Comparison	HR	95% CI	*p* Value
Age	Continuous (per year increase)	1.036	1.003–1.070	0.031
**Gender**	Male vs. Female	0.97	0.454–2.071	0.937
**Liver disease**	Yes vs. No	1.376	0.411–4.604	0.605
**COPD**	Yes vs. No	1.181	0.491–2.844	0.710
**Cardiovascular disease**	Yes vs. No	2.353	0.979–5.658	0.076
**Kidney disease**	Yes vs. No	0.0	0–INF	0.998
**Type II diabetes**	Yes vs. No	1.095	0.411–2.916	0.855
**Previous cancer**	Yes vs. No	2.105	0.49–9.044	0.317
**WHO2022 diagnosis**	Secondary AML vs. De Novo AML	2.953	1.361–6.408	0.006
***NPM* status**	Positive vs. Negative	0.173	0.122–1.027	0.074
***FLT3* status**	Positive vs. Negative	1.353	0.122–1.027	0.004
**ELN2022 risk**	High vs. Low/Intermediate	4.184	1.899–9.215	<0.001
**Chemotherapy regimen**	HMA/Ven vs. Intensive	2.124	0.952–4.743	0.036
**Disease status**	Refractory vs. Responding	4.590	2.067–10.192	0.017
**PAP**	Caspofungin vs. Posaconazole	1.23	0.568–2.662	0.559
**Neutropenia duration**	Continuous (per day increase)	1.032	1.005–1.058	0.019
**HSCT**	Yes vs. No	0.334	0.126–0.884	0.027
**IFI**	Proven/Probable vs. None	3.032	1.023–8.985	0.045
**Multivariate Analysis**				
**Age**	Continuous (per year increase)	1.15	1.054–1.254	0.002
**Disease status**	Refractory vs. Responding	5.009	1.503–11.695	0.009

COPD = chronic obstructive pulmonary disease; WHO = World Health Organization; ELN = European Leukemia Net; PAP = primary antifungal prophylaxis; HSCT = hematopoietic stem cell transplantation; IFI = invasive fungal infection.

## Data Availability

Data are provided within the manuscript or in the Supplementary Materials file.

## Supplementary Materials

The following supporting information can be downloaded at https://www.mdpi.com/article/10.3390/cancers17132184/s1, Supplemental Results (S1): Correlation analysis with Spearman’s rank ρ correlation test; Supplemental Results (S2): Univariate Logistic Regression for possible, probable and proven IFI (With Firth correction) results; Supplemental Results (S3): Univariate Logistic Regression only for probable and proven IFIs (With Firth correction) results; Supplemental Results (S4): Multivariate Logistic Regression only for probable and proven IFIS with Firth correction and enter method for covariate interaction; Supplemental Results (S5): Overall Survival according in refractory patients according to type of prophylaxis (Caspofungin vs. Posaconazole), with log-rank test results.
