# Supplementary material for: Caspofungin for Primary Antifungal Prophylaxis in Acute Myeloid Leukemia: A Real-Life Study from an Academic Center
Source: Cancers (Basel). 2025 Jun 28;17(13):2184. doi: 10.3390/cancers17132184 (PMC12248434; doi:10.3390/cancers17132184)

CASPOFUNGIN FOR PRIMARY ANTIFUNGAL PROPHYLAXIS IN ACUTE MYELOID LEUKEMIA: A REAL-LIFE STUDY FROM AN ACADEMIC CENTER

Supplementary results

Supplemental results 1 (S1).

Correlation analysis with Spearman's rank ρ correlation test

**Liver disease**	0.373
**Neutropenia days**	0.269
**Refractory to chemotherapy**	0.257
**HMA/Ven therapy**	0.162
**COPD**	0.150
**Caspofungin**	0.126
**Age**	0.076
**NPM/FLT3 positive**	0.069
**Cardiovascular**	0.043
**Secondary AML**	0.033
**ELN2022 risk**	0.011
**Diabetes II**	-0.025
**Kidney**	-0.116
**Previous Cancer**	-0.116
**Gender**	-0.131

Supplemental results 2 (S2).

Univariate Logistic Regression (With Firth correction) results.

Variable of Outcome: IFI Proven / Probable / Possible

Variabile	Comparison	OR	Est.	std.err	CI95_low	CI95_up	P
**gender**	Male vs Female	0,37	0,44	0,73	0,05	2,26	0,28
**age**	Continous	0,97	1,01	0,03	0,89	1,05	0,43
**liver**	Yes vs No	10,48	10,17	0,83	1,58	237,43	0,01
**copd**	Yes vs No	2,08	2,67	0,78	0,14	32,08	0,58
**cardiovascular**	Yes vs No	0,49	1,37	0,86	0,00	7,26	0,64
**kidney**	Yes vs No	0,07	0,44	0,00	0,00	3,67	0,27
**diabetes**	Yes vs No	1,13	0,83	0,84	0,06	13,24	0,92
**previous_cancer**	Yes vs No	0,97	0,44	0,00	0,00	50,64	0,99
**WHO2022**	Secondary vs De Novo AML	2,27	1,22	0,70	0,10	627,77	0,62
**NPM**	Positive vs Negative	1,05	1,30	0,70	0,07	9,75	0,97
**FLT3**	Positive vs Negative	0,68	1,31	0,75	0,06	6,52	0,74
**ELN2022risk**	High Vs Int/low	0,23	1,14	0,69	0,00	5,41	0,37
**chemotherapy**	HMA/VEN vs Intensive	3,38	2,67	0,72	0,14	435,92	0,48
**Disease status after CHT**	Refractory Vs Responding	4,44	4,75	0,75	0,70	82,29	0,12
**neutropenia_days**	Continous	1,00	1,01	0,01	0,97	1,04	0,78
**antifungal prophylaxis**	antifungalCaspofungin	2,67	2,11	0,69	0,54	31,97	0,23

Supplemental results 3 (S3).

Univariate Logistic Regression (With Firth correction) results.

Variable of Outcome: IFI Proven AND Probable (Possible excluded)

Variable	Comparison	OR	Est.	std.err	CI95_low	CI95_up	P
**gender**	Male vs Female	-0,98	0,42	0,87	0,06	2,12	0,32
**age**	Continous	-1,00	0,97	0,03	0,92	1,03	0,32
**liver**	Yes vs No	2,51	9,45	0,89	1,53	56,79	0,01
**copd**	Yes vs No	-0,13	0,86	1,13	0,04	5,79	0,90
**cardiovascular**	Yes vs No	-0,13	0,86	1,13	0,04	5,79	0,90
**kidney**	Yes vs No	1,01	0,64		0,00	6,44	0,76
**diabetes**	Yes vs No	-0,55	0,54	1,12	0,03	3,51	0,58
**previous_cancer**	Yes vs No	1,01	0,64		0,00	6,44	0,76
**WHO2022**	Secondary vs De Novo AML	-1,18	0,27	1,11	0,01	1,70	0,24
**NPM**	Positive vs Negative	1,27	2,79	0,81	0,57	15,19	0,20
**FLT3**	Positive vs Negative	1,09	2,44	0,82	0,44	12,21	0,27
**ELN2022risk**	High Vs Int/low	-1,24	0,25	1,11	0,01	1,60	0,22
**chemotherapy**	HMA/VEN vs Intensive	-0,55	0,54	1,12	0,03	3,51	0,58
**Disease status after CHT**	Refractory Vs Responding	1,89	4,92	0,84	0,86	26,17	0,05
**neutropenia_days**	Continous	1,01	1,01	0,01	0,98	1,04	0,03
**antifungal prophylaxis**	Caspofungin Vs Posaconazole	0,73	1,79	0,80	0,37	9,69	0,47

Supplemental results 4 (S4).

Multivariate Logistic Regression with Firth correction and enter method for covariate interaction

Variable of Outcome: IFI Proven AND Probable (Possible excluded)

Variable	OR	CI_lower	CI_upper	p_value
**gender**	0,767	0,002	9,917	0,816
**age**	0,96	0,83	1,039	0,299
**liver**	30,491	2,654	2,664E+15	0,004
**copd**	1,009	0	27,286	0,996
**cardiovascular**	24,52	0,044	46114740,6	0,249
**kidney**	2,223	0,001	370,845	0,731
**diabetes**	1,419	0,069	30,824	0,81
**previous_cancer**	23,633	0,036	1596797,53	0,236
**WHO2022**	1,757	0,026	2091,243	0,769
**NPM**	2,098	0,218	160,305	0,499
**FLT3**	0,327	0	3,743	0,379
**ELN2022risk**	0,051	0	4,382	0,156
**chemotherapy**	0,756	0,001	45,984	0,893
**Disease status after CHT**	11,968	1,204	1023,689	0,033
**neutropenia_days**	1,013	0,976	1,297	0,494
**antifungal prophylaxis**	1,663	0,226	3149,759	0,583

Supplemental results 5 (S5).

Overall Survival according in refractory patients according to type of prophylaxis (Caspofungin vs Posaconazole), with log-rank test results

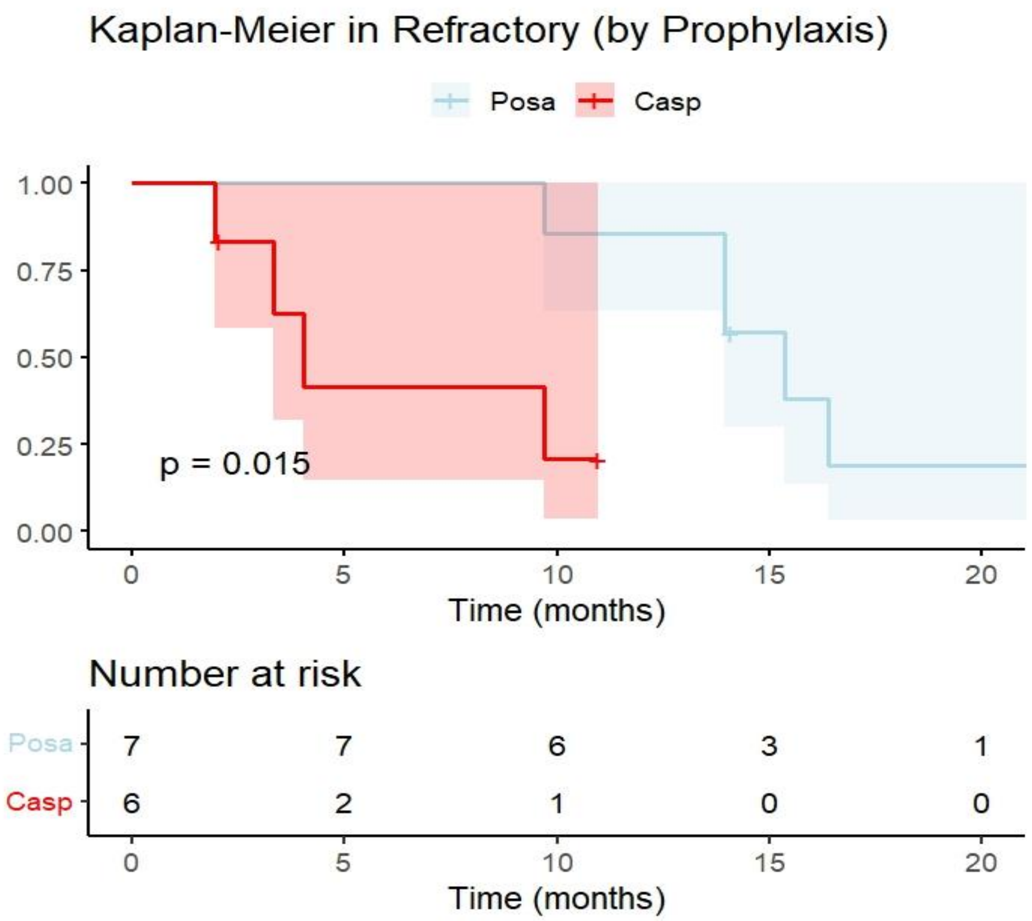

Supplement: Supplementary file 1 [file cancers-17-02184-s001.zip › cancers-3696574-supplementary.pdf]
